# Uncovering Forensic Taphonomic Agents: Animal Scavenging in the European Context

**DOI:** 10.3390/biology11040601

**Published:** 2022-04-15

**Authors:** Lara Indra, David Errickson, Alexandria Young, Sandra Lösch

**Affiliations:** 1 Department of Physical Anthropology, Institute of Forensic Medicine Bern, University of Bern, 3008 Bern, Switzerland; sandra.loesch@irm.unibe.ch; 2 Cranfield Forensic Institute, Cranfield University, Bedford MK43 0AL, UK; david.errickson@cranfield.ac.uk; 3 Independent Researcher, Bournemouth BH12 5BB, UK; youngaforensicarch@me.com

**Keywords:** forensic anthropology, scavenging, taphonomy, bone lesions, tooth marks

## Abstract

**Simple Summary:**

In forensic cases of exposed human bodies, vertebrate animals frequently pose challenges concerning remains recovery, identification, and the interpretation of circumstances of death. For instance, they can remove body parts, destroy skeletal features, and create “pseudo-lesions” that complicate trauma analysis. With this review, we highlight vertebrate scavengers in Europe that are of forensic relevance, including their typical behaviour and their inflicted lesions on bones and soft tissues. Here, we support forensic practitioners in recognising animal activity at the site of discovery and in interpreting the circumstances of death. Our review serves as a guide for the examination of animal-altered human remains and their found state by providing ecological backgrounds on various vertebrate species. In addition, this study provides an overview of the current state of research in the field of animal scavenging in forensics.

**Abstract:**

Animal scavenging by vertebrates can significantly alter human bodies and their deposition site. For instance, vertebrate animals can cause postmortem modification to a body, alter perimortem trauma, influence decomposition rates, disarticulate and scatter body parts or evidence, and affect the identification of the deceased. Animal scavenging is a relatively common occurrence in forensic investigations. Even so, studies on the subject are scattered and rare, with most focussing on geographical areas outside of Europe. For that reason, we intend to collate the literature to provide an account of forensically relevant vertebrate scavengers in Europe, their impacts on human remains, and their implications for forensic investigations. Here, we provide an overview of forensic aspects where the knowledge of animal scavenging is crucial, as well as an account of potential scavengers of human remains in Europe and their typical alterations to soft tissue and, in particular, to bones. In addition, we are the first to provide a guide for forensic practitioners to identify the presence of vertebrate scavenging and subsequently inform outdoor search strategies for affected human remains.

## 1. Introduction

Upon the discovery of human remains, it is imperative that the scene examiner can identify taphonomic processes that may have altered their appearance. In particular, this includes animal activity in outdoor settings. For example, animals can significantly affect the human remains and the area of deposition. The severity and duration of animal scavenging, as well as animal succession, depend on various factors, including temperature, time of day, season, environment, and distance to human settlements nearby [1,2,3,4]. Animals may feed on soft tissue or bones and disarticulate body parts. In these cases, the knowledge of species-specific vertebrate scavenger behaviour can be helpful in interpreting the outdoor forensic scene. For instance, an understanding of scavenger actions and their consequences can aid a forensic investigation with the accurate reconstruction of circumstances surrounding death [5,6,7]. In addition, information about vertebrate activity may support the investigation regarding the search and recovery of human remains, improved accuracies of the postmortem interval (PMI) or the length of exposure estimates, and distinguishing actual person-inflicted trauma from scavenging-induced postmortem damage [8,9].

Vertebrate modifications to human remains are frequently encountered [9,10,11,12]. For instance, Komar [10] analysed 596 cases in New Mexico and reported that postmortem animal activity was detected in 46% (*n* = 162). Furthermore, of 714 reviewed cases in North America, 15% (*n* = 107) mentioned vertebrate animal scavenging on human bones [13]. Additionally, from 22 forensic outdoor cases in Switzerland, 31.8% (*n* = 7) exhibited lesions created by vertebrate scavengers [12]. Furthermore, a conference abstract presents scavenging rates in 107 forensic cases from Southern Nevada of 28% (*n* = 30) [14]. Young et al. [11] surveyed 111 police specialist searchers in the UK and found that 63% of them knew scenes where scavenging had affected human remains. These few forensic publications show that vertebrate scavenging is an underestimated aspect in the field, yet its potential for influencing interpretations is vast. Nevertheless, studies on scavenging in forensic contexts are limited to certain regions and cannot directly be applied to other geographical contexts. 

For instance, previous studies either focus on species not endemic in Europe, e.g., lions [15,16,17,18] and hyenas [15,19], or the species are also endemic in Europe, but the studies were placed on other continents, e.g., vulture studies in the US [20,21,22]. In the latter, comparable data from other regions or from captive environments should only be applied with critical reflection. This is because species adapt to certain environmental conditions, and their behaviour can differ in this regard [22,23]. 

Therefore, this article categorises the potential vertebrate scavengers of human remains in outdoor settings within mainland Europe and its islands. For this, we collated the literature from multiple research areas, including ecology, taphonomy, and biology. We intend this article to serve as a guide when examining an outdoor forensic scene yielding animal-altered remains. Specifically, our work is aimed at forensic practitioners in Europe by providing an overview of the vertebrates that are known to alter bodies or forensic scenes. In addition, we have categorised information on species-typical behaviour and species-characteristic modifications to soft tissue and, in particular, bone. With this work, the readers become informed about the impact vertebrate fauna can have on human remains in a European context. The aim is to acquaint forensic practitioners with findings at a scene associated with animal activity, advising search strategies and preventing the misinterpretation of evidence.

## 2. Implications of Vertebrate Scavenging

Vertebrate-inflicted modifications to human remains can have various implications for forensic analyses. First, several species are known to begin their feeding at orifices and wounds due to the easier access to inner structures [24,25,26]. By feeding at traumatic lesions, animals may alter or obliterate information that might be used to identify the cause of death or interpret violence-related injuries [5,24,25,27,28]. On the other hand, scavenging creates new lesions and damage through actions related to the consumption of tissue, possibly mimicking trauma or mutilation to the body. Reports show that dogs can decapitate human bodies and consume the entire head in the process [29,30], sea lice can create skin lesions looking like shotgun pellets [28], and carnivores may inflict wounds looking like sharp-force trauma or gunshot injuries [8,31,32]. In other cases, animal claw marks were misinterpreted as defence scratches made by human fingernails [33], and rodent-inflicted marks were seen to resemble violent human actions [34,35,36].

Second, animal scavenging can cause issues in interpreting the rate of decomposition and subsequently in the estimation of time since death. The consumption of soft tissue accelerates the decomposition process [2,28,37,38,39,40,41,42], while the consumption of insects and the prevention of their oviposition can slow down the decomposition rate [5,43,44,45]. If scavenging activity goes unnoticed, applied methods may lead to false PMI estimates, especially when they rely on decomposition stages, insect activity, or the chemical properties of the underlying soil [4,5,38,42]. However, in some cases, vertebrate scavengers can also provide approximate information on the time since death. For example, specific animals will normally visit a corpse within their preferred stage of decomposition or during a certain environmental setting, e.g., winter. For instance, rodents attracted to dry bone were noted as the most promising animals to aid PMI estimation because of their late involvement, usually at 30 months postmortem or later [13,46].

Additionally, animal scavenging marks on bone can leave traces that might be mistaken for other taphonomic variables, for instance, beak or talon scores resembling the etching of the bone surface by plant roots [20].

Last, some animals are able to disarticulate body parts or skeletal elements completely. They may scatter and transport them away from the initial deposition site, usually to bring them to their offspring or to feed in a more protected environment [47,48,49]. This causes challenges in the search and recovery phase of an investigation. However, recovering the entire body is important in terms of identification and the interpretation of the cause of death [47,48,50]. Especially in forests, searchers should be acquainted with recognising game trails, as well as the nests and burrows of potential scavenging agents [9]. In an experiment, Young et al. [51] showed that providing information about the scavenging behaviour of the red fox prior to the search of a mock scatter scene significantly enhances the recovery efforts.

## 3. Forensically Important Vertebrate Scavengers in Mainland Europe and the UK

Although it is difficult to identify the scavenger species of a body without direct observation, there are some ways to do so. For instance, this can be carried out by assessing the environment and geography [7], the scavenging pattern on a body or skeletal element [18,21,52], tooth mark type and dimensions on bone [53,54,55,56], and associated faunal evidence such as scats, feathers, or fur [35,57]. In addition, molecular genetic analyses can be applied to identify the creator of certain lesions [58].

Tooth mark definitions generally follow the widely adopted categories suggested by Binford in 1981 [59]: pit, puncture, score, and furrows. Pits and punctures are created by tooth tips; while the pit is only a depression, a puncture perforates the bone surface. By dragging teeth over bone, scores and furrows may be formed. Scores are linear, shallow striations, whereas furrows are deeper, penetrating grooves, usually reaching into the spongiosa. Furrows may also be observed inside the long bone ends, where scavengers “gouged” out the trabeculae to access bone marrow.

### 3.1. Felids (Order Carnivora, Family Felidae)

In Europe, two species of small cats are free-ranging, namely house cats (*Felis catus*) and wild cats (*Felis silvestris*). Whereas domestic cats are numerous and found near human settlements, wild cats are mainly endemic in specific regions of the Iberian Peninsula, France, Italy, the Balkans, and the Scottish Highlands [60,61]. In some parts of Europe but not in the UK, larger felid species can be found: the Eurasian lynx (*Lynx lynx*) and the Iberian lynx (*L. pardinus*), the latter only in Spain and Portugal [62]. Lynx prefer woodland to live and prey in and can hunt animals up to their own size. Cats are carnivorous, and although they prefer to prey and occasionally scavenge smaller animals [63,64], there are also reports of human remains scavenged by cats [65,66,67,68,69,70]. 

**Figure 1:**
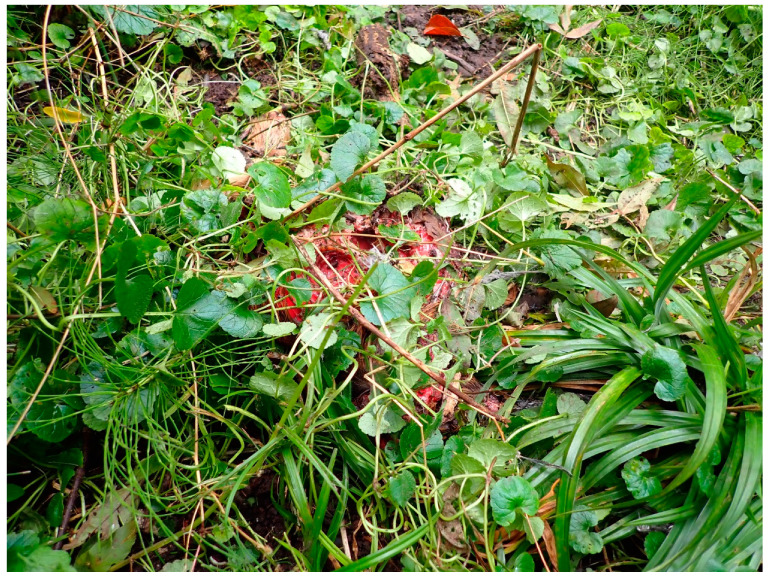
Parts of a deer carcass covered with plants by a Eurasian lynx in an experimental setting in Switzerland (Tierpark Bern).

Cats prefer to hunt and eat their prey, but if scavenging, they mainly scavenge on fresh carcasses [65,67]. They will feed over the course of several days taking breaks in-between [33,67]. Although small cats are unlikely to transport entire human bodies, they can remove and scatter body parts, especially when skeletonised [66,71]. As part of this act, cats may also cache the body (parts) by placing them in natural depressions (no digging) and covering them with vegetation (Figure 1) or even the body’s hair [33,72,73,74]. 

Domestic cats primarily scavenge from the face, head, neck, upper limbs, and hands [65,68,70]. Feral domestic cats seem to start with areas where lesions are present and then progress to feed on the upper limbs and the chest [67]. Byard [68] showed that a clowder of cats inflicted extensive damage to the hands, face, neck, thorax, and inner organs. Cats tend to start feeding on fatty tissue followed by muscles, reflecting the skin layers and potentially damaging the bone in the course of doing so. Their teeth cause large defects in the tissue, with smaller, irregular defects peripheral to the main scavenging area [67,68]. In an experiment with a human body, lynx fed on the soft tissue of the lower arms, the hips, and upper thigh region [33]. In addition, cats can create linear scratches and circular punctures by inserting their claws into the skin to stabilise the movement of the limb or surrounding tissue when feeding [33,67]. Interestingly, legs seem to be the least affected area of cat scavenging [67,68]. 

**Figure 2:**
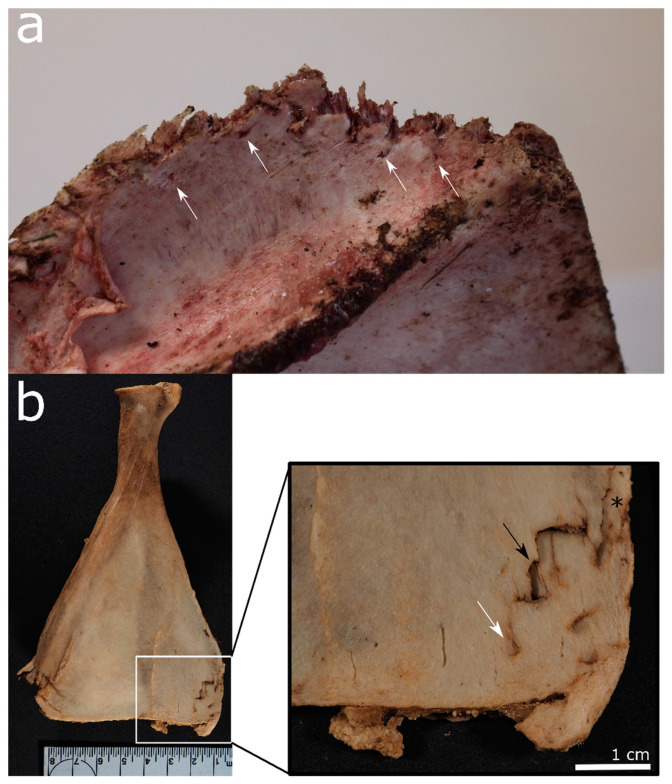
Animal bones scavenged by felids: (**a**) Distal edge of a scapula scavenged by a cheetah in an experimental setting in the UK. Note the irregular margins and narrow pits alongside (white arrows). (**b**) A deer *scapula* gnawed by Eurasian lynx in an experimental setting in Switzerland (Tierpark Bern). Solely the distal edges show pits (white arrow), punctures (black arrow), and a combination of punctures and crushing (asterisk).

On bone, cats create pits, punctures, grooves, and scores. However, cats are not generally interested in consuming bone, and bone damage usually occurs as a by-product of soft tissue scavenging. For example, lynx exposed a human radius and ulna by feeding off the soft tissue around them, only leaving marks in the distal ulna and a metacarpal bone [33]. Cat-inflicted punctures are often paired due to the felid’s distinctive dental morphology of the lower first molars [66,75]. Furthermore, felid punctures are particularly deep and narrow, especially in comparison to canid tooth marks [75]. As demonstrated by Moran and O’Connor [75], these tooth marks are normally found in a concentrated area or as isolated pits and punctures (Figure 2).

Álvarez et al. [76] examined rabbit (leporid) bones scavenged by the small felid Geoffroy’s cat (*Leopardus geoffroyi*) and found that 20% of the bones exhibited tooth marks, mainly pits and punctures, followed by crenulated edges, scoring, and furrows, as well as spiral fractures. However, when evaluating larger human bones, it is likely that only the smaller skeletal elements would exhibit similar findings [7]. Garcia et al. [67] examined fresh human bodies scavenged by feral cats in an outdoor setting and noted no damage to the bones at all, only to the soft tissue.

### 3.2. Canids (Order: Carnivora, Family: Canidae)

Domestic dogs (*Canis domesticus*), red foxes (*Vulpes vulpes*), raccoon dogs (*Nyctereutes procyonoides*), and the grey wolf (*Canis lupus*) are the abundant canids in Europe, while the latter two are not endemic in the UK. Dogs are generally bound to humans, whereas foxes inhabit almost all kinds of terrain in the UK [77]. Although per definition, carnivores, *Canidae,* are facultative carnivores and can therefore process plant-based food. Domestic dogs appear as scavengers in the taphonomic literature in both outdoor settings [19,27,78,79,80,81,82] and indoor contexts [28,65,70,83,84,85,86,87,88,89]. Similarly, scavenging studies of the red fox are common [1,90,91,92], but most have less of a forensic focus [2,19,81,93,94]. 

The scavenging sequence of canids differs between species and whether they scavenge indoors or outside. Outdoors, the most widely used sequence was established by Haglund et al. (1989) [95]. In their study from the Pacific Northwest, coyotes and domestic dogs scavenged 22 of 30 human bodies. They demonstrated that canids consumed soft tissue-rich areas first and then disarticulated upper and lower limbs until only the vertebral column remained articulated. Following this, scattering of the bones took place. The disarticulation sequence of wolves studied by Willey and Snyder [5] yielded similar results: wolves started feeding on meaty regions of the lower limbs, then the thoracic cavity and throat, and the vertebral column was fed upon last. Disarticulation of the limbs generally occurs within the first 48 h. In an indoor environment, canids seem to focus on the cranial and neck regions [88]. On human-sized animal carcasses, red foxes start scavenging at the hind limbs, then continue on to the thorax and finally the skull [56]. Hewson and Kolb (1976) [90] reported that red foxes do not consume guts and, on average, eat 1 kg of meat per day. Pack-scavenging wolves may consume fawns (16–20 kg) entirely within 24 h and deer (55–73 kg) within four to seven days [5]. Although red foxes will feed throughout all stages of decomposition, they prefer to feed on the carrion in the early stages [80,96], and in the colder seasons the red fox will increase their scavenging frequency [43].

Canids are scatter hoarders, thus caching their food in many different places [73,97]. Red foxes may dig holes of up to 12 cm in depth to hide food and then cover it with vegetation and soil, sometimes scent-marking it with urine [96]. Canids will move scavenged body parts from the initial deposition site to scavenge away from the carcass without competition, to feed offspring in a den, or cache food for later consumption [5,56,98,99,100]. Haglund et al. [95] observed domestic dogs transporting a human mandible approximately 400 m away, and Young et al. [56] reported that a red fox scattered deer remains up to a distance of 103 m. In a study with deer carcasses, wolves immediately started to drag away the carcass, and in this process, a fawn body was even wrapped around a sapling [5]. However, the bones in this study were distributed wildly rather than being reaccumulated in a single place. Generally, wolves cache if they are sated, mainly during summer months, and may do so with entire small-sized bodies or regurgitated small amounts of food by either covering it with natural material or by placing it into dug up shallow holes [101].

On the soft tissue, canids leave uneven, crenulated wound margins with V-shaped punctures. Similarly, there are often skin lacerations and soft tissue avulsions from tearing skin and flesh [30,84,95]. From the canid’s claws, four to five parallel scratches on the skin may be identifiable [30,102]. Often, the complete skeletonisation of the skull and neck (and sometimes the upper thorax, too) is recorded, especially indoors. In these cases, the dogs removed and consumed only the soft tissue. Furthermore, “hole-and-tear injuries” are characteristics created by domestic dogs where canine punctures are coupled with adjacent skin tears. This is created by shaking while holding the body or soft tissue in its mouth [102]. In some cases, dogs removed entire body parts, including hands, fingers, or feet [88]. 

Skeletal damage usually occurs when canids gnaw through bones in an attempt to enter the thorax, abdominal cavity, or medullary cavity. As a result, canids typically create pits, punctures, scores, furrows, spiral fractures, depressed fractures, bone splintering, flaking at the bone edges, the entire removal of the epiphyses, and subsequently, the “scooping out” of the shaft ends [59,99]. Of these characteristics, pits are the most abundant [56], but the most typical canid bone modification is the destruction of long bone epiphyses. This is achieved in an attempt to reach the bone marrow, leaving cylindric long bone shafts with crenulated, uneven edges, which are sometimes smoothened due to licking into the shaft [49,59,99].

**Figure 3:**
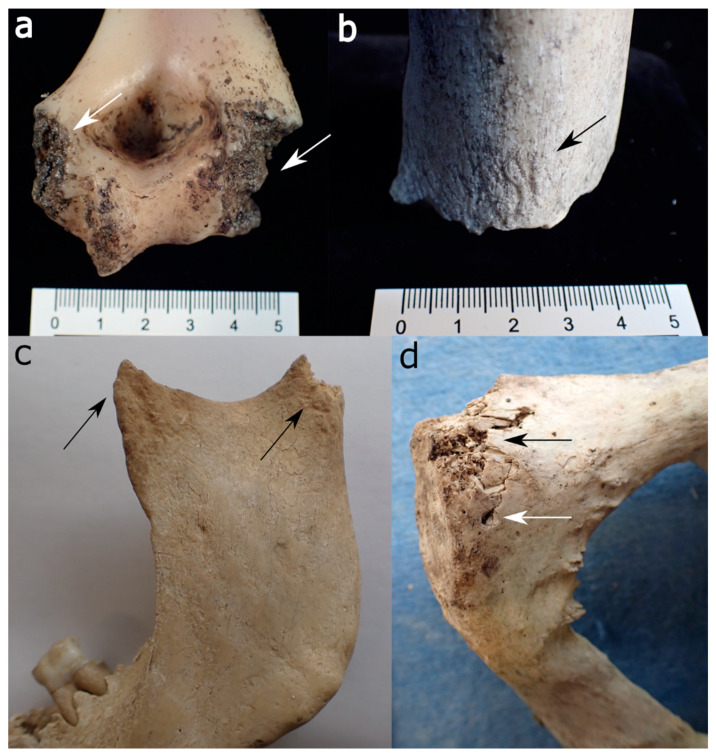
Canid modifications on human bones from forensic casework: (**a**) Gnawing damage on protruding parts of the distal humerus epiphysis (white arrows). (**b**) Uneven, crenulated margin, smoothing of the edges and scoring on the outer surface (black arrow) of a distal femur. (**c**) Removal of condylar head, pits, and scores on the coronoid and condylar process of a mandible (black arrows). (**d**) Gnawing damage (black arrow) and isolated punctures (white arrow) on the posterior aspect of a pubic bone.

Bones gnawed by canids are often highly fragmented, especially as they may partake in “boredom chewing” [59]. The regions on the human skeletons that commonly exhibit postmortem damage include protruding elements of the skull and mandible (mastoid processes, nasal bones, mandibular ramii, etc.), the upper parts and margins of the scapula, the transverse and spinous processes of the vertebrae, the sacrum, the pubic symphysis and iliac crest of the pelvis, the sternal ends of the ribs, and the trabecula-rich ends of long bones, e.g., [5,86,98,103,104]. Figure 3 show long and irregular bone examples exhibiting canid scavenging. Medium-sized canids can also cause spiral fractures of smaller and medium-sized bones, e.g., human lower arm bones and fibulae [99]. 

Finally, if bones are excreted or regurgitated by canids, they may present polishing, perforations, corrosion, and smoothened edges due to gastric acid [105,106].

### 3.3. Ursids (Order: Carnivora, Family: Ursidae)

Out of eight bear species, only one is freely ranging in parts of Europe, the brown bear (*Ursus arctos*). Brown bears live in heavily wooded areas, mainly in the north and southeast of Europe, and they are not (anymore) endemic in the UK. Although bears belong to the order *Carnivora*, their diet is omnivorous and includes plants, insects, fish, preyed mammals, as well as occasionally carrion [62].

In a pig cadaver experiment, they were the first scavengers and also the ones that produced the most extensive damage to carcasses [107]. Bears further tend to remove the carcass or parts of it from its initial deposition site and can transport it up to several hundred meters away to consume and cache it, although they generally do not bring the remains into their dens [107,108]. Scattering of the remains commonly occurs from the secondary location [108]. Elgmork (1982) found that brown bear caching sites in Norway are between 3.5 to 75 m^2^ large and likely situated in small forest clearings on flat ground that is not associated with human infrastructure. The sites are usually raked by the bear prior to deposition and coverage of the cadaver with vegetation, and often, bear scats, beds with bear fur, and bitten trees are found nearby, indicating that they tend to guard it [109]. 

On pig cadavers, bears scavenged first on the soft tissue of the limbs, thorax and head, organs following the skin, and muscles [107]. Bright [107] further notes that the sequence might differ with animals that have a different fat distribution or with the season and the hibernation cycle of bears. 

**Figure 4:**
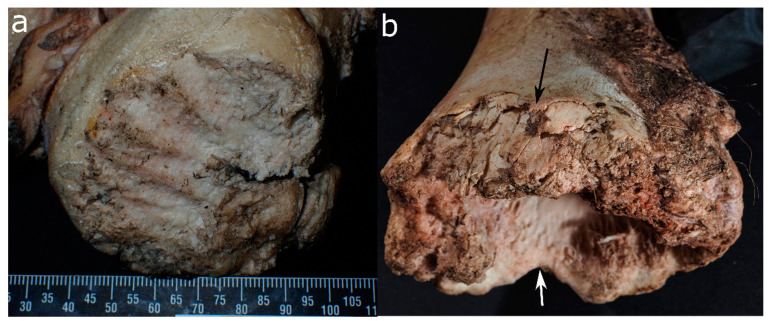
Cow bones gnawed by a brown bear in Switzerland (Tierpark Goldau): (**a**) condyle of the femur with large furrows and (**b**) gouged out end of a long bone with irregular and crushed margins (both arrows) that in some places appear rounded (white arrow).

**Figure 5:**
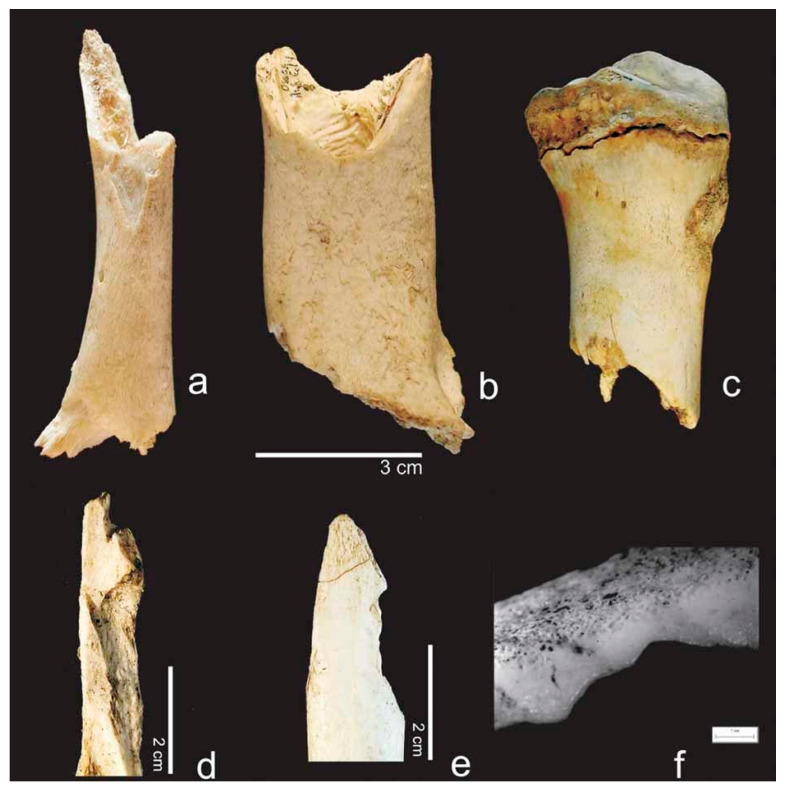
Damage to bones caused by brown bears: (**a**–**c**) removal of one or both epiphyses, (**d**,**e**) chipped back edges, (**f**) licking on a breakage plane. Reproduced with permission from Saladié et al. [110]; published by John Wiley & Sons Ltd, 2013.

On bone, they produce similar damage to other large carnivores [110,111]. Nevertheless, some features are deemed characteristic of bears. For instance, Carson et al. (2000) found that they preferably exploit the axillary skeleton (vertebral column and rib cage) while canids concentrate on the extremities and inner organs [111]. However, this finding was not confirmed by a pig cadaver study, where bears preferred limbs over the axial skeleton [107]. Udoni [112] further notes that their scavenging produces a comparably high amount of scooping and scalloping on the epiphyses of large long bones. Human skeletons scavenged by bears further showed the following damage: damage to the maxilla, temporal bones, and palate; the removal of spinal and transverse processes of the vertebrae; damage to the pelvis, including the iliac crest, ischial tuberosity, and the pubic bone; spiral fractures of small and large long bones; and the removal of epiphyses from large limb bones (Figure 4 and Figure 5). Additionally, they state a concentration of tooth pits and punctures near the edge of long bone shaft margins [111]. Bear-inflicted scores can be deep and parallel as well as shallow and random, and they have a U-shaped cross-section often associated with crushed cortical bone [110].

### 3.4. Mustelids (Order: Carnivora, Family: Mustelinae)

Several mustelid species are endemic in Europe, including the UK, such as the weasel (*Mustela nivalis*), stoat (*Mustela erminea*), American mink (*Mustela vison*), ferret (*Mustela furo*), polecat (*Mustela putorius*), European pine marten (*Marten marten*), otter (*Lutra lutra*), wolverine (*Gulo gulo*), and Eurasian badger (*Meles meles*) [63,113,114]. Mustelids are, as per their definition, carnivores, although their diet varies between species, including strict carnivores such as weasels and martens and omnivores such as badgers [62]. Although some mustelids are opportunistic scavengers [26,91,115], only the Eurasian badger, American mink, fisher (*Martes pennanti*), and stoat appear in forensic literature [81,96,116,117,118,119]. This is surprising as mustelids are generally widespread, and the badger (*Meles* sp.) and wolverine belong to the larger wild scavengers of exposed remains [56,120]. 

Mustelids prefer to scavenge on the unclothed areas of the body, and badger and mink activity increases with advancing decomposition [96,116]. Small mustelids such as fisher are further reported to consume flesh from around the anus and bullet holes because entering through existing body openings is easier [26]. Mustelids are caching animals; thus, they may remove body parts, scatter and/or transport them away from the deposition site, and/or cover remains with natural materials [26,96]. In order to consume from a covered carcass, fisher was observed doing both, either freeing the carcass from the caching material or penetrating it with their muzzle [26].

Mustelids seem to prefer the rear parts of carrion, as shown for pig and bear carcasses scavenged by mink and American marten, respectively [26,116]. However, there are no detailed descriptions of soft tissue lesions inflicted by mustelids.

**Figure 6:**
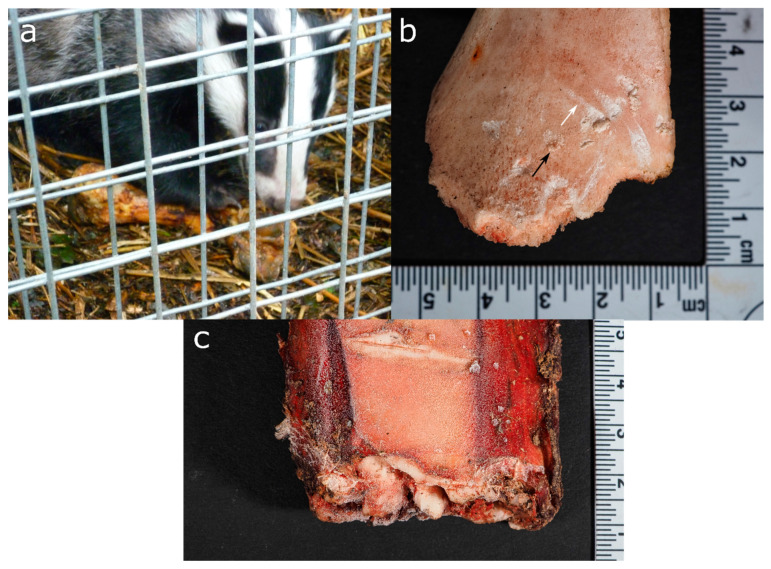
Scavenging experiments (from UK and Switzerland) with captive Eurasian badgers demonstrate (**a**) holding of a pig long bone with its claws and (**b**) removing of the epiphysis of a large herbivore long bone shaft, leaving an uneven, crenulated margin with pits (black arrow) and scores (white arrow), (**c**) a bovine rib end with irregular margins and crushed bone.

Some studies showed that pits are the most common tooth marks by mustelids encountered in bone, followed by scores and a few punctures [56,118]. Badgers hold their food steady or tear soft tissue with their claws (Figure 6). Therefore, claw marks may possibly be identified [121]. Extensively gnawed long bone epiphyses with uneven margins and sometimes scooping, similar to canid scavenging, are also found [56,118]. Although, the damage pattern resembles that from canids, due to the stronger bite force of Eurasian badgers compared to red foxes, their tooth marks are usually larger than fox-inflicted marks [122]. Interestingly, studies of American mink scavenging partially submerged pig carcasses demonstrated that they only consumed soft tissue without damaging the bone [116,119].

### 3.5. Procyonids (Order: Carnivora, Family: Procyonidae)

Raccoons (*Procyon lotor*) were introduced to Europe in the early twentieth century, and escapees started freely ranging populations, now covering areas in Central and Eastern Europe [63]. However, there are currently no wild raccoons in the UK. They live in deciduous forests or under bush vegetation near flowing water, but also in more open areas and near settlements. They use either natural cavities or abandoned dens to sleep in [63].

Raccoons are omnivores; their diet includes, for example, berries, nuts, mussels, small rodents and birds, eggs, and also carrion [63]. An experimental study comparing scavenging on human, pig, and rabbit bodies reported that raccoons show a strong preference for human tissue and only scavenged pigs in winter [4]. Furthermore, raccoons were observed to partially remove a body bag from human corpses prior to feeding [46].

**Figure 7:**
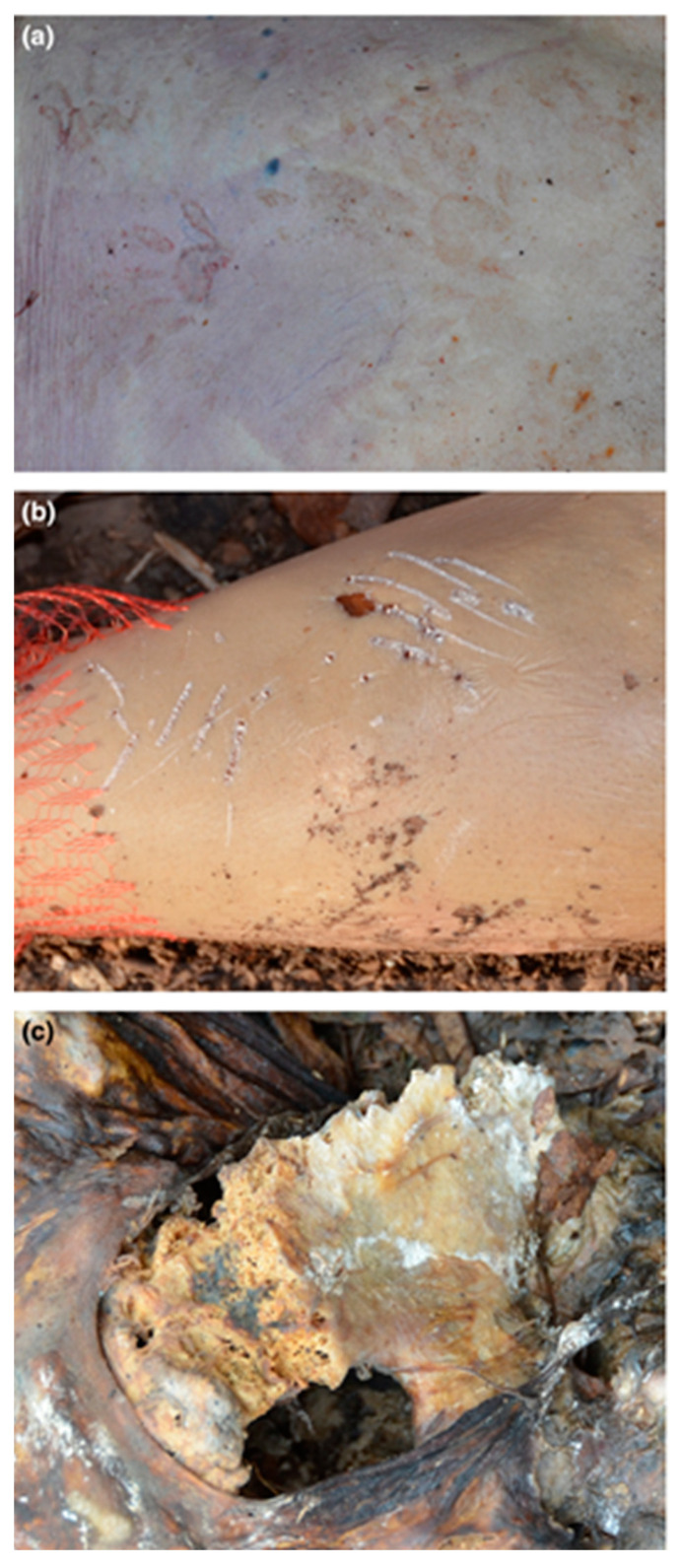
Experimental raccoon-scavenged human remains showing (**a**) paw prints on the back skin, (**b**) scratch marks on a leg and (**c**) gnawing damage to an innominate bone. Reproduced with permission from Jeong et al. [123]; published by Elsevier, 2016.

In an experimental setting, raccoons were more likely to and faster at starting to scavenge human remains in summer than winter, but with extended scavenging periods in cold months (over 30 days on average) compared to warmer months (less than 10 days on average) [123]. The same study found that they started scavenging during the early stages of decomposition in 75% (72 out of 96) of the human cases, and in non-human cases, their scavenging was limited to fresh tissues [123]. Synstelien [45] reported a sequence of raccoon behaviour at a human corpse as follows: feeding on soft tissue, feeding on maggot masses, collecting maggots, and digging for insect pupae in the soil. Pawprints may be found on the skin, as shown in Figure 7a.

Primary access by raccoons to the human bodies starts at limbs, mainly by opening the skin via small, oval perforations. Further locations include the feet, face, and genitalia, followed by the abdomen [4]. Lower limbs were scavenged more frequently, and before upper limbs, the head was scavenged least frequently [45]. Raccoons may also “play” with a body as seen with animal cadavers, e.g., pulling out the tongue, intestines, or fur [4]. Disarticulation of body parts may occur in the course of their scavenging as well as some scattering, although raccoons were not observed to transport bones away [45].

The soft tissue scavenging of raccoons showed a unique pattern by causing an oval hole into the human skin and then extracting muscle tissue through this hole with their forepaws, leaving the skin collapsed and increasing the chance of mummification [4,123]. Synstelien [45] reported patchy epidermal lesions, especially on foot and ankle regions, as well as nipped-off tissues at the distal ends of fingers, palms, toes, and soles, including nails. The author further showed raccoons removing the scalp of two bodies in later decomposition stages, defleshing the face, or feeding on the muscles of foot soles. Furthermore, they are likely to leave parallel scratch marks on the skin due to climbing or clutching the body (Figure 7b) [45,123].

Racoons create bone modifications in the course of soft tissue scavenging rather than due to the aim of bone consumption. Damaged bones are mostly found as crushed and splintered distal pedal and manual phalanges up to carpals and tarsals. Shallow scores perpendicular to the axis of long bones can also be found, as well as furrows and other damage along epiphyseal margins, e.g., at the tibial plateau or ilium (Figure 7c) [45]. However, in one pig experiment with raccoon scavenging, no bone lesions were detected [124].

### 3.6. Suids (Order: Artiodactyla, Family: Suidae)

Suid species include domestic pigs (*Sus scrofa domesticus*) and wild boars (*Sus scrofa*). The latter are adaptive and inhabit a wide range of environments in Europe and the UK [61].

**Figure 8:**
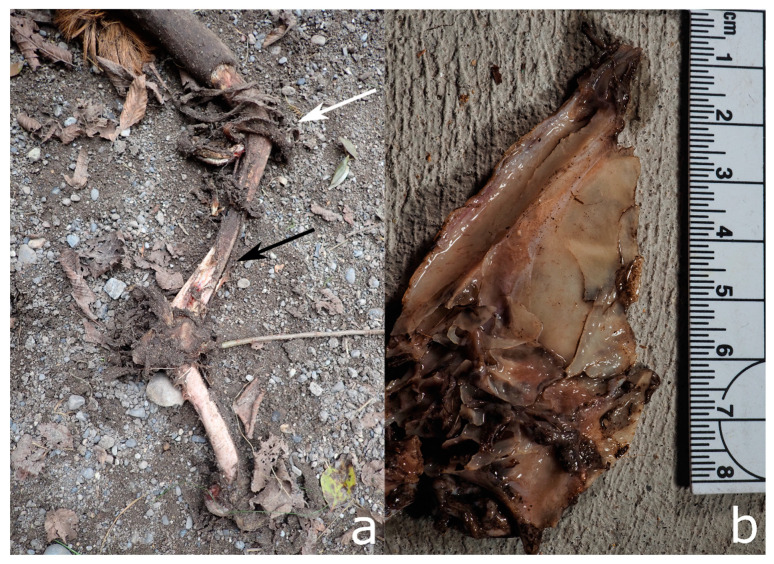
Wild boar-impacted bones from an experimental setting in Switzerland (Tierpark Bern): (**a**) Deer leg with detached skin due to trampling. Note the dirt-covered soft tissue still adherent (white arrow) and the spiral fracture (black arrow). (**b**) Remains of a deer skull with crushed and fractured margins.

The omnivorous pigs scavenge on carrion of various sizes [19,79,80,94,125,126,127], especially in autumn and winter [128]. Wild boars are able to smell food even underground and will dig it up [127]. To deflesh bones, pigs either trample on them or use their lower incisors to scrape off the meat (Figure 8) [125,129]. On human bodies, suids tend to focus on the anterior body midline, including the skull, thorax, and pelvis [125], while wild boars prefer damaged fresh bone and are known to revisit carrion repeatedly. Domínguez-Solera and Domínguez-Rodrigo [130] observed that suid species do not scatter skeletal elements more than about four metres.

Although there are limited publications regarding pig-inflicted marks on soft tissue, some studies focus on bone. For instance, Berryman [125] demonstrated that bone damage was concentrated in the thorax and pelvic region. Suids create broad and shallow scores on the bone with their flat, lower incisors. These marks are different to the V-shaped deeper scores created by canids [49,125,129,130]. Though scores are the main characteristics, isolated punctures and outer damage of long bone epiphyses may occur [125,130].

Suids usually destroy and swallow smaller skeletal elements; medium-sized ones exhibit extensive damage and fragmentation, while larger elements remain more or less intact [129,130].

### 3.7. Rodents (Order: Rodentia)

Rodent species populate almost all habitats in Europe and the UK; they include rats and mice (*Muridae*), dormice (*Gliridae*), squirrels (*Sciuridae*), beavers (*Castoridae*), and voles (*Cricetidae*). Different rodent families have various feeding habits; some of them are omnivores, while others are herbivores or mainly live on insects. Omnivorous rodents such as rats will feed on soft tissue throughout all decomposition stages [34,35,45,131,132]. For mineral intake or incisors shortening, rodents may scavenge on dry or fresh bone, even if they are herbivorous [46,49]. However, Klippel and Synstelien [46] found that squirrels rarely gnaw on bone with a PMI of less than 30 months. In addition to rodents, shrews (*Soricidae*), moles (*Talpidae*), and hedgehogs (*Erinaceinae*), as well as rabbits and hares (*Lagomorpha*), are also widespread in Europe, including the UK. Lagomorphs are occasional scavenging agents and can be observed, in rare cases, to feed on fresh animal carcasses and bone [91,133,134]. 

Small bones and body parts are frequently transported away from the deposition site to the rodent’s burrows or nests. This allows the animal to cache and feed safely [37,73]. For example, squirrels are documented to remove medium-sized bones, such as a human clavicle or cattle ribs, from the deposition site and cache them [46,127]. In a study by Klippel and Synstelien (2007), grey squirrels frequently removed bones from the study site (ca. 8000 m^2^) and only once was a bone recovered on the ground, 12 m uphill from its initial position [46]. In some instances, rodents use desiccated human bodies as a latrine, shelter, or even build a nest within, allowing the body to further serve as overhead protection for burrow entrances beneath it [35,45,57]. 

**Figure 9:**
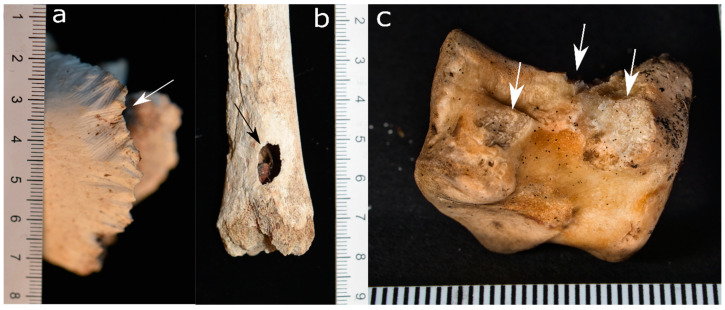
Experimental rodent modifications on animal bones: (**a**) Parallel striations caused by incisors dragging over the bone margins, arranged in fan-shaped patterns (white arrow). (**b**) A “window” created by incisors scraping over the same spot multiple times (black arrow), as modification by captive *Degu octodon*. (**c**) Nibbled off edges (white arrows) by mice in a Swiss forest. Only the left lesion exhibits the rodent-typical striations.

Soft tissue scavenging usually occurs on unclothed body parts, while other coverings such as plastic bags may be ripped open [34,35,84,131,135]. Although rodents may, on occasion, feed on the associated insects [136] and on the body itself, they initially concentrate on locations of penetrating trauma [24]. Rats were observed to be most interested in fatty tissue, undermining the skin, but also consuming skin and muscle tissues [45]. Rodents further initiate their scavenging at exposed areas of the face and hands [34,35,84,131,135] (Figure 9a), sometimes including the trimming of finger- or toenails [70]. Typically, rodents create layered, tight, and circumscribed defects with both relatively smooth or otherwise scalloped margins and no or few alterations beyond this margin [35,45,135]. Additionally, brown rats were seen to leave impressions of their claws in the skin and upon them scavenging on desiccated soft tissue, the remnants appeared shredded and frayed [45]. 

Bone lesions created by rodents are frequent. On relatively fresh bone, rodents preferably gnaw on cancellous-rich areas with thin cortical layers. In the process, they may “pedestal” even large long bones, e.g., a distal femur, leaving only some bone connecting the epiphyses to the shaft [45,135]. Furthermore, rats considerably damage the distal bones of the hands and feet [45]. With their incisors, rodents create parallel or fan-shaped striations at a bone’s margins or protuberances (e.g., supraorbital margin or mandible) and, likewise, parallel striations perpendicular to the long bone axis [35,49]. Figure 9b illustrate breakthrough “windows” in long bone shafts, created by incisors gnawing at one place [45,121]. Figure 9c show the nibbed off edges of a spongiosa-rich pig bone. 

### 3.8. Cervids and Bovids (Order: Artiodactyla, Family: Cervidae, Bovidae)

In Europe, cervids (*Cervidae sp.*) and bovids (*Bovidae sp.*) are widely distributed. They include ungulate (hooved) species such as cattle (*Bos sp.*), goats (*Capra sp.*), sheep (*Ovis sp.*), and in the UK specifically, six deer species (*Cervus elaphus* and *C. nippon*, *Capreolus capreolus*, *Dama dama*, *Muntiacus reevesi*, *Hydropotes inermis*). All of them need grassland or open meadows to feed, but some species such as the Chinese water deer, are good swimmers and live near water sources [127]. Cervids and bovids populate the entire UK, including most islands around the mainland [61]. In the rest of Europe, deer include some further species, such as chamois (*Rupicapra rupicapra*), Alpine ibex (*Capra ibex*), elk (*Alces alces*), reindeer (*Rangifer tarandus*), and chital (*Axis axis*) [127].

These herbivore animals do not typically consume soft tissue [127] and case studies with forensic relevance focussing on cervids [137,138] and bovids [139] are limited. Similarly, to the author’s knowledge, the only record given for ungulates gnawing human bone is from a white-tailed deer (*Odocoileus virginianus*) in a research facility in Texas [140]. In a pig-cadaver study in South Africa, a domestic cow herd rolled over the carcass a few times, but this was not recorded as scavenging [136].

**Figure 10:**
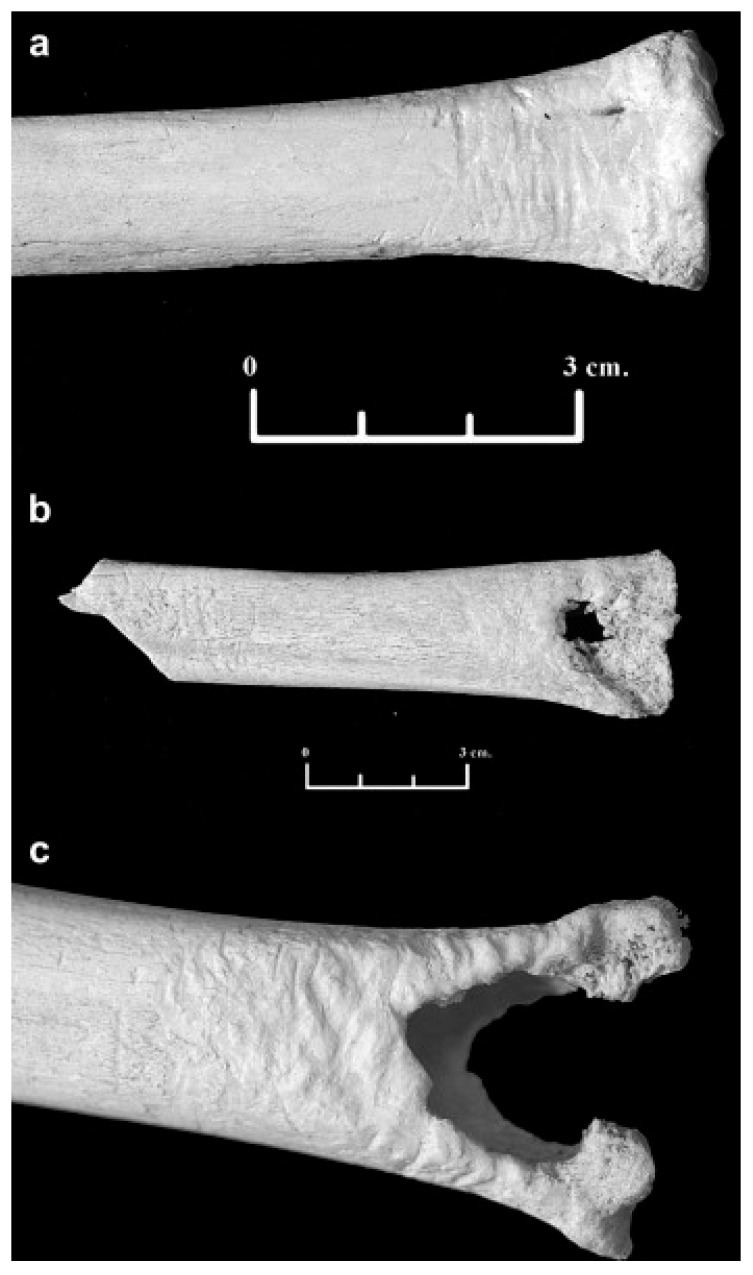
Animal bones gnawed by ungulates in an experiment: (**a**) transversal grooves, (**b**) exposed trabeculae and disappearing epiphysis, (**c**) fork-shaped distal long bone. Reproduced with permission from Cáceres et al. [141]; published by Elsevier, 2011.

Ungulates may chew on dry bone (osteophagia) to source phosphorous and possibly calcium due to a deficiency [141,142]. Therefore, ungulate manipulations take place in the later decomposition stages, when bone is dry already. Typically, their gnawing marks are likely to be present on bones with a high proportion of compacta relative to spongiosa and long and flat bones, e.g., diaphyses [141]. Often, this presents as a fork-like shape with a wavy pattern along its ends. Bone margins may also be rounded due to the saliva from repeated chewing in the same position [137,142]. Ungulates chewing damage on dry bone might superimpose weathering cracks [141]. Figure 10 present different appearances of bones chewed by ungulates.

Hooves of ungulates may further damage bone by trampling or wallowing, especially when grains are present in the underlying substrate [143,144,145]. 

### 3.9. Birds (Class: Aves)

More than 500 bird species breed in Europe, of which over 200 also inhabit the UK. Some are frequent scavengers, such as vultures (*Accipitridae*), corvids (*Corvidae*, e.g., ravens and crows *Corvus sp*., and magpies *Pica sp.*), various birds of prey (e.g., eagles, buzzards and hawks *Accipitridae*, and owls *Strigidae* and *Tytonidae*), and seabirds such as gulls (*Laridae*). However, many avian species are known to sporadically scavenge or alter a forensic scene but are not or are seldom reported in the forensic literature. For example, the red kite (*Milvus milvus*), pigeons (*Columbiformes*), pheasants (*Phasianidae*), and various water birds such as ducks (*Anatidae*). 

Most taphonomic studies found birds to be persistent scavengers and, on occasion, the earliest carrion visitors [1,25,48,91,146,147]. However, many studies concentrate on particular species such as vultures [15,20,21,22,72,148,149,150,151] and eagles [1,94,152,153,154]. Vulture species in Europe include the bearded vulture (*Gypaetus barbatus*), cinereous vulture (*Aegypius monachus*), Egyptian vulture (*Neophron percnopterus*), in Southern Europe, the African Rüppell’s vulture (*Gyps rueppelli*), and the most common species in Europe, the griffon vulture (*Gyps fulvus*).

The time of arrival of birds at a carcass depends on the species and the decomposition stage. Buzzards (*Buteo buteo*) are only observed to scavenge prior to the bloating stage [24], whereas carrion crows (*Corvus corone*) are present throughout the entire decomposition [2,3,24]. If lesions are present, corvids and buzzards will start scavenging there [24,25,26]. If there is no penetrating trauma, birds may wait for other animals to open the carcass [26,155]. Birds are also able to transport body parts or associated evidence (e.g., bones, jewellery, and hair) for several hundred metres [2,24,25]. The bones can even be dispersed through the airstream while taking off and landing [25]. In particular, corvids are known to cache food away from the deposition site [146]. Many birds might be more attracted to the insects colonising a body rather than feeding on the body. Thus, potentially altering PMI estimations based on entomofauna [3,24,45,93,125,136,148].

**Figure 11:**
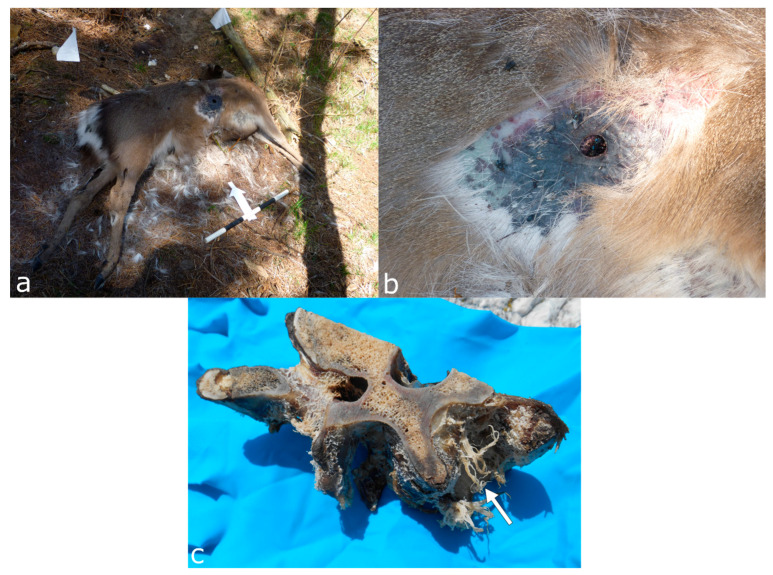
Crows removed the fur around the gunshot wound of a deer carcass in a forensic experiment in the UK, (**a**) overview and (**b**) close-up. (**c**) A bovine vertebra scavenged by seagulls; note the frayed soft tissue remains (white arrow). Figure 11c reproduced with permission from Pokines [159]; published by John Wiley & Sons Ltd, 2022.

Corvids, buzzards, and hawks remove string-like strips with straight edges by pecking and tearing soft tissue with their beaks [24,25,147,156]. These lesions can resemble cut marks. Similarly, conical or V-shaped punctures localised in the thorax and abdomen region are created by the animal’s beak [25,155]. In an experiment with deer carcasses, crows removed fur from around a gunshot wound, leaving a bald patch (Figure 11) [157]. In other instances, songbirds and seabirds created patchy epidermal lesions [70,158]. After consuming the soft tissue, corvids may leave loose remains of nerves, tendons, and ligaments attached to the joints [156].

**Figure 12:**
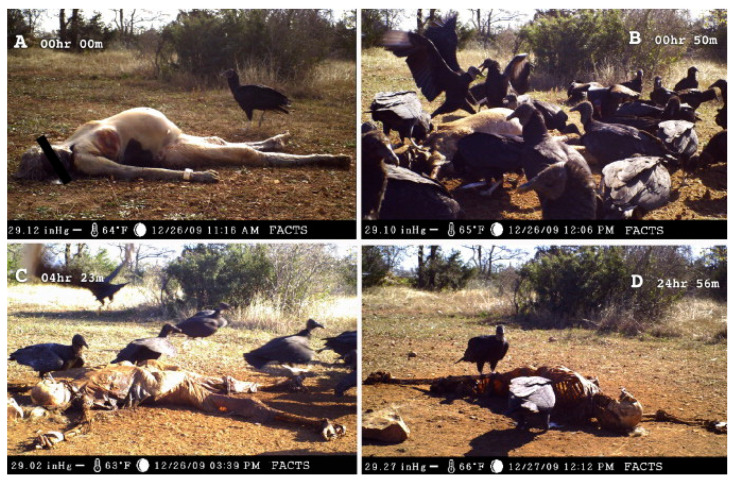
Vultures scavenging donated human remains at the Forensic Anthropology Research Facility (FARF) in Texas. The images show the remains at deposition (**A**), after one hour (**B**), after over four hours (**C**), and after ca. 24 h (**D**). Note the largely skeletonised body, with the bones preserved in anatomical order through soft tissue remnants. Reproduced with permission from Spradley et al. [21]; published by Elsevier, 2012.

Birds often aim for soft tissue rather than bone, which can lead to almost entirely skeletonised bodies with the bones still articulated through soft tissue remains in anatomical order [20,21,156]. A group of vultures, for instance, can fully skeletonise an adult human-sized body in the course of hours [20,21,150,160] (Figure 12). Birds may try to access the bone marrow or leave pits, punctures, and scores on bone as a by-product of using their beaks and talons as tools for soft tissue scavenging [20,153]. The linear marks left on bone by beaks or talons are usually unpaired or clustered in a roughly parallel manner or coupled and forming a V- or L-shape [149]. Vultures, for example, can leave linear but irregularly shaped scores of several centimetres in length, more similar to root etching than sharp force trauma [20]. Additionally, conical or V-shaped pits and punctures, as well as notches along the margins of flat or broken bones, can occur upon bird scavenging [25,145]. So-called “can-opener” perforations of thin bones, presenting with surrounding bone flaps, were observed on monkey remains scavenged by crowned hawk-eagles [153,154]. Bone digested by birds usually shows acid etching, causing the dissolution and weakening of the bone surface [145,154].

## 4. Discussion

Our paper is the first to collate comprehensive data on most European vertebrate scavengers with a focus on implications for forensic practice, including species-specific modifications on soft tissue and bone. The review is supposed to aid the initial assessment of a scene and further evaluation of findings, such as lesions or scattering. It further supports decisions on the inclusion of taphonomy experts.

The literature review demonstrates the underestimated relevance of animal activity at body scenes, as well as the lack of forensic studies on animal scavenging, in particular in Europe. Although species-specific as well as particular environment studies are important for reconstructions in outdoor forensic settings, there is an imbalance of scavenger taxa and geographical distribution in the literature. Most publications concentrate on locations within the US and, subsequently, their endemic animal taxa. For example, birds were casually mentioned as scavengers in many research and case reports [78,82,161]. However, detailed publications on avian scavengers preferably cover vultures [20,21,22,148,150,151], which are rare in most of Europe and not endemic in the UK. To apply evidence about taphonomic, vertebrate inflicted lesions in forensic casework in Europe, the relevant species must be prior studied regarding their modifications to soft and bone tissue.

Our article highlights that data outcomes vary, even if the studies examined similar topics. Contradicting results are especially problematic regarding evidence in forensic investigations, and practitioners should be aware of the variety of outcomes. These differences are partially due to various environmental settings but also due to inconsistencies in study designs encompassing the carcasses, accessibility for scavengers, duration, recording, and documentation. Some factors depend on the legal framework of the countries where the research is conducted. For example, taphonomic research on human bodies is illicit in most countries and so far only implemented in the US, Canada, Australia, and the Netherlands [162,163]. In addition, the carcasses differ in pre-experimental treatments, such as to cause of death or freeze-storage. However, blood loss, clothing, and freezing are shown to affect scavengers and decomposition [164,165,166]. Additionally, the scavengers studied may entail divergences even within the same species due to environment-adaptive behaviour [23], as shown for wild and captive Eurasian badgers and pumas [96,167]. For these reasons, existing studies should be revised to verify, falsify, or adjust previous results. In doing so, a standardised approach for the documentation of animal altered remains should be implemented, including inter alia measurement techniques of tooth marks on bone. A consistent methodology would improve comparability between studies, environments, carcasses, and species. 

Scavenging vertebrate species show overlaps in their behaviour, tooth morphology, inflicted lesions, and tooth marks. For example, circular tooth punctures can be attributed to different scavengers. Therefore, identifying a scavenger species just from the scavenged remains is difficult. However, some scavenging alterations are more likely to be inflicted by certain taxa. We compiled scavenger alterations to human remains and their attribution to the aforementioned animal taxa (Table 1).

A common challenge in forensic anthropology is to distinguish between bone lesions caused by human-inflicted trauma and those caused by taphonomic influences, such as scavenging [28,168]. One reason is that scavenging often occurs on biomechanically fresh bone, impeding the separation of peri- and postmortem damage [169]. Another explanation is that scattering in outdoor casework frequently prevents the full recovery of the remains, and the missing bones cannot be interpreted [10,12,50,170]. For a solid interpretation, it is crucial to analyse the lesion itself, the pattern of damages, and to include the environmental context of the recovery scene.

In the following, we compiled the most important aspects when assessing the cause of bone lesions in forensic cases with potential animal involvement. Further details can be found in the relevant sections on animals above.

- Location and pattern of lesions. Animal scavenging can result in species-typical damage to the skeleton. Observed damage to human remains from forensic casework can be compared to published damage patterns.
- Lesion morphology. The manifestations of trauma caused by sharp force are well described in the literature [171]. Additionally, for many animal species, there are reports on typical lesions, such as the tooth marks of carnivores [54,55,99,112] and rodents [35,49] that are not usually confused with trauma. However, tooth marks may be concentrated in areas consumed or removed from the scene by the scavengers, and in the absence of tooth marks, it is difficult to attribute non-specific force impacts such as fractures to either trauma or scavenging [168]. In forensic casework, it is important to observe as many skeletal elements as possible as well as to include environmental factors in the analysis. Observations will then be compared with known and reported causes.
- Lesion surrounding. The immediate surrounding of a lesion can provide information about the processes that caused the damage. For instance, if long bone epiphyses are broken off, a scavenger cause is more likely than a traumatic cause if there are “gouged out” shaft ends, pits and punctures, and smoothing of the lesion edges due to extensive animal licking [49,59,99].
- Lack of vital reactions. No haemorrhaging at a wound indicates a postmortem cause, which is by definition the case with scavenging. On weathered bones, taphonomically induced lesions are often lighter in colour than the surrounding bone [168]. However, perpetrators can also carry out postmortem mutilations, often involving dismemberment of the body or concealment of identity.
- Direct evidence of scavengers. Sometimes, vertebrate scavengers are observed on the corpse itself or nearby, which makes them a likely or even certain scavenger of the remains [172]. It is further possible to install camera traps at the site to capture returning vertebrates, even after the remains are removed.
- Indirect evidence of scavengers. Animal activity often leaves other traces on or nearby the body. For instance, scats and droppings, regurgitate, feathers and hair, nests, burrows and beds, gnawed vegetation, footprints, etc. [20,35,57,109,123].

## 5. Conclusions

Animal scavenging as a taphonomic factor alters human remains at forensic scenes, especially outdoors. By consuming tissue, creating lesions, disarticulating the body, and transporting and caching parts, vertebrate scavengers add further complexity to a forensic investigation. Although studies and case reviews are published, they concentrate on certain geographical regions and species that are not endemic in Europe. Therefore, it is paramount that we conduct further research on animal scavenging in forensic contexts, specifically within Europe and the UK.

Our article should serve as a tool for forensic practitioners who are engaged in outdoor environments. The provided information can aid in the search, recovery, interpretation, and identification of human remains altered by vertebrate scavengers. Future work should comprise analyses of vertebrate scavenger behaviour, disarticulation sequences, scatter patterns, and species-typical inflicted marks on soft and hard tissue. Within this article, we collected the data published so far, pointing out missing information and potential in a forensic context. Therefore, this paper serves as a foundation for further vertebrate scavenging studies.

## Figures and Tables

**Table 1:** Potential European vertebrate scavenger taxa of outdoor forensic scenes and their characteristic modifications on human tissue. X = typically present, (x) = may be present, - = typically not present.

	Felid	Canid	Ursid	Mustelid	Procyonid	Suid	Rodent	Cervid/Bovid	Birds
Behaviour									
Soft tissue consumption	X	X	X	X	X	X	X	-	X
Bone consumption	(x)	X	X	X	(x)	X	X	X	(x)
Transport	X	X	X	X	-	-	X	-	X
Caching	X	X	-	X	-	-	X	-	X
Trampling	-	-	-	-	-	X	-	X	-
Bone modifications									
Claw marks	X	X	X	X	X	-	-	-	X
Conical pits	X	X	X	X	(x)	-	-	-	-
Irregular pits	X	X	X	X	(x)	X	-	-	X
Punctures	X	X	X	X	(x)	(x)	-	-	X
Scores	X	X	X	X	X	X	-	X	X
Furrows	X	X	X	X	X	-	-	-	-
Epiphyseal removal	(x)	X	X	X	-	X	-	(x)	(x)
Scooping	-	X	X	X	-	-	-	-	-
Crenulated edges	X	X	X	X	(x)	X	-	-	-
Spiral fractures	(x)	X	X	(x)	-	(x)	-	-	-
Splintering	X	X	X	X	X	X	-	X	-
High fragmentation	-	X	-	X	-	X	-	X	-
Pedestalling	-	-	-	-	-	-	X	-	-
Windows	-	-	-	-	-	-	X	-	-
Small, parallel striations	-	-	-	-	-	-	X	X	-
Notches along border	-	-	-	-	-	-	-	-	X

## Data Availability

Not applicable.

## References

[1] Read J.L., Wilson D. (2004). Scavengers and Detritivores of Kangaroo Harvest Offcuts in Arid Australia. Wildl. Res..

[2] O’Brien R.C., Forbes S.L., Meyer J., Dadour I. (2010). Forensically Significant Scavenging Guilds in the Southwest of Western Australia. Forensic Sci. Int..

[3] O’Brien R.C., Forbes S.L., Meyer J., Dadour I.R. (2007). A Preliminary Investigation into the Scavenging Activity on Pig Carcasses in Western Australia. Forensic Sci. Med. Pathol..

[4] Steadman D.W., Dautartas A., Kenyhercz M.W., Jantz L.M., Mundorff A., Vidoli G.M. (2018). Differential Scavenging among Pig, Rabbit, and Human Subjects. J. Forensic Sci..

[5] Willey P.L., Snyder M. (1989). Canid modification of human remains—Implications for time-since-death estimations. J. Forensic Sci..

[6] Ubelaker D.H., Sorg M.H., Haglund W.D. (1997). Taphonomic applications in forensic anthropology. Forensic Taphonomy: The Postmortem Fate of Human Remains.

[7] Sincerbox S.N., DiGangi E. (2017). Forensic Taphonomy and Ecology of North American Scavengers.

[8] Rodriguez W.C., Sorg M.H., Haglund W.D. (1997). Decomposition of buried and submerged bodies. Forensic Taphonomy: The Postmortem Fate of Human Remains.

[9] Nawrocki S.P., Blau S., Ubelaker D.H. (2016). Forensic taphonomy. Handbook of Forensic Anthropology and Archaeology.

[10] Komar D.A. (2003). Twenty-Seven Years of Forensic Anthropology Casework in New Mexico. J. Forensic Sci..

[11] Young A., Stillman R., Smith M.J., Korstjens A.H. (2014). Scavenging in Northwestern Europe: A Survey of UK Police Specialist Search Officers. Policing.

[12] Indra L., Lösch S. (2021). Forensic Anthropology Casework from Switzerland (Bern): Taphonomic Implications for the Future. Forensic Sci. Int. Rep..

[13] Ubelaker D.H., DeGaglia C.M. (2020). The Impact of Scavenging: Perspective from Casework in Forensic Anthropology. Forensic Sci. Res..

[14] Woollen K., Byrnes J.F. A Retrospective Analysis of Scavenging in Southern Nevada Forensic Anthropology Cases (2000–2021). Proceedings of the American Academy of Forensic Sciences 74th Annual Scientific Conference.

[15] Blumenschine R.J. (1976). Carcass consumption sequences and the archaeological distinction of scavenging and hunting. J. Hum. Evol..

[16] Dominguez-Rodrigo M. (1999). Flesh availability and bone modifications in carcasses consumed by lions. PALAEO.

[17] Domínguez-Rodrigo M., Gidna A.O., Yravedra J., Musiba C. (2012). A comparative neo-taphonomic study of felids, hyaenids and canids—An analogical framework based on long bone modification patterns. J. Taphon..

[18] Parkinson J.A., Plummer T., Hartstone-Rose A. (2015). Characterizing Felid Tooth Marking and Gross Bone Damage Patterns Using gis Image Analysis: An Experimental Feeding Study with Large Felids. J. Hum. Evol..

[19] Lotan E. (2000). Feeding the Scavengers. Actualistic Taphonomy in the Jordan Valley, Israel. Int. J. Osteoarchaeol..

[20] Reeves N.M. (2009). Taphonomic Effects of Vulture Scavenging. J. Forensic Sci..

[21] Spradley M.K., Hamilton M.D., Giordano A. (2012). Spatial Patterning of Vulture Scavenged Human Remains. Forensic Sci. Int..

[22] Dabbs G.R., Martin D.C. (2013). Geographic Variation in the Taphonomic Effect of Vulture Scavenging: The Case for Southern Illinois. J. Forensic Sci..

[23] McPhee M.E., Carlstead K., Kleiman D.G., Thompson K.V., Baer C.K. (2010). The importance of maintaining natural behaviors in captive mammals. Wild Mammals in Captivity: Principles and Techniques for Zoo Management.

[24] Young A., Stillman R., Smith M.J., Korstjens A. (2014). An Experimental Study of Vertebrate Scavenging Behavior in a Northwest European Woodland Context. J. Forensic Sci..

[25] Komar D., Beattie O. (1998). Identifying bird scavenging in fleshed and dry remains. Can. Soc. Forensic Sci. J..

[26] Hüner E.A., Peter J.F.B. (2012). In Situ Caching of a Large Mammal Carcass by a Fisher, Martes Pennanti. Can. Field-Nat..

[27] Rothschild M.A., Schneider V. (1997). On the temporal onset of postmortem animal scavenging. “Motivation”-of the animal. Forensic Sci. Int..

[28] Byard R.W., James R.A., Gilbert J.D. (2002). Diagnostic problems associated with cadaveric trauma from animal activity. Am. J. Forensic Med. Pathol..

[29] Buschmann C., Solarino B., Püschel K., Czubaiko F., Heinze S., Tsokos M. (2011). Post-mortem decapitation by domestic dogs: Three case reports and review of the literature. Forensic Sci. Med. Pathol..

[30] Tsokos M., Byard R.W., Puschel K. (2007). Extensive and Mutilating Craniofacial Trauma Involving Defleshing and Decapitation: Unusual Features of Fatal Dog Attacks in the Young. Am. J. Forensic Med. Pathol..

[31] Puskas C.M., Rumney D.T. (2003). Bilateral fractures of the coronoid processes. Differential diagnosis of intra-oral gunshot trauma and scavenging using a sheep crania model. J. Forensic Sci..

[32] Symes S.A., Williams J.A., Murray E.A., Hoffman J.M., Holland T.D., Saul J.M., Saul F.P., Pope E.J., Sorg M.H., Haglund W.D. (2002). Taphonomic context of sharp-force trauma in suspected cases of human mutilation and dismemberment. Advances in Forensic Taphonomy: Method, Theory, and Archaeological Perspectives.

[33] Rippley A., Larison N.C., Moss K.E., Kelly J.D., Bytheway J.A. (2012). Scavenging Behavior of Lynx Rufus on Human Remains during the Winter Months of Southeast Texas. J. Forensic Sci..

[34] Patel F. (1994). Artefact in forensic medicine—Postmortem rodent activity. J. Forensic Sci..

[35] Haglund W.D. (1992). Contribution of rodents to postmortem artifacts of bone and soft tissue. J. Forensic Sci..

[36] Prahlow J.A., Linch C.A. (2000). A baby, a virus, and a rat. Am. J. Forensic Med. Pathol..

[37] Mann R.W., Bass W.M., Meadows L. (1990). Time since death and decomposition of the human body. Variables and observations in case and experimental field studies. J. Forensic Sci..

[38] Buchan M.J., Anderson G.S. (2001). Time since death: A review of the current status of methods used in the later postmortem interval. Can. Soc. Forensic Sci. J..

[39] Suckling J.K., Spradley M.K., Godde K. (2016). A Longitudinal Study on Human Outdoor Decomposition in Central Texas. J. Forensic Sci..

[40] Galloway A., Sorg M.H., Haglund W.D. (1997). The Process of Decomposition: A Model from the Arizona Sonoran Desert. Forensic Taphonomy: The Postmortem Fate of Human Remains.

[41] Sorg M.H., Dearborn J.H., Monahan E.I., Ryan H.F., Sweeney K.G., David E., Sorg M.H., Haglund W.D. (1997). Forensic Taphonomy in Marine Contexts. Forensic Taphonomy: The Postmortem Fate of Human Remains.

[42] Giles S.B., Harrison K., Errickson D., Márquez-Grant N. (2020). The Effect of Seasonality on the Application of Accumulated Degree-Days to Estimate the Early Post-Mortem Interval. Forensic Sci. Int..

[43] Bass W.M., Sorg M.H., Haglund W.D. (1997). Outdoor Decomposition Rates in Tennessee. Forensic Taphonomy: The Postmortem Fait of Human Remains.

[44] Dillon L.E., Anderson G.S. (1996). Forensic Entomology: A Database of Insect Succession on Carrion in Northern and Interior BC.

[45] Synstelien J.A. (2015). Studies in Taphonomy: Bone and Soft Tissue Modifications by Postmortem Scavengers. Ph.D. Thesis.

[46] Klippel W.E., Synstelien J.A. (2007). Rodents as Taphonomic Agents: Bone Gnawing by Brown Rats and Gray Squirrels. J. Forensic Sci..

[47] VanLaerhoven S.L., Hughes C. (2008). Testing different search methods for recovering scattered and scavenged remains. Can. Soc. Forensic Sci. J..

[48] Kjorlien Y.P., Beattie O.B., Peterson A.E. (2009). Scavenging activity can produce predictable patterns in surface skeletal remains scattering: Observations and comments from two experiments. Forensic Sci. Int..

[49] Pokines J.T., Pokines J.T., Symes S.A. (2013). Faunal Dispersal, Reconcentration, and Gnawing Damage to Bone in Terrestrial Environments. Manual of Forensic Taphonomy.

[50] Komar D.A., Potter W.E. (2007). Percentage of body recovered and its effect on identification rates and cause and manner of death determination. J. Forensic Sci..

[51] Young A., Stillman R., Smith M.J., Korstjens A.H. (2016). Applying Knowledge of Species-Typical Scavenging Behavior to the Search and Recovery of Mammalian Skeletal Remains. J. Forensic Sci..

[52] Parkinson J.A., Plummer T.W., Bose R. (2014). A gis-based approach to documenting large canid damage to bones. Palaeogeogr. Palaeoclimatol. Palaeoecol..

[53] Coard R. (2007). Ascertaining an agent: Using tooth pit data to determine the carnivore/s responsible for predation in cases of suspected big cat kills in an upland area of britain. J. Archaeol. Sci..

[54] Delaney-Rivera C., Plummer T.W., Hodgson J.A., Forrest F., Hertel F., Oliver J.S. (2009). Pits and pitfalls: Taxonomic variability and patterning in tooth mark dimensions. J. Archaeol. Sci..

[55] Andrés M., Gidna A.O., Yravedra J., Domínguez-Rodrigo M. (2012). A study of dimensional differences of tooth marks (pits and scores) on bones modified by small and large carnivores. Archaeol. Anthropol. Sci..

[56] Young A., Stillman R., Smith M.J., Korstjens A.H. (2015). Scavenger Species-Typical Alteration to Bone: Using Bite Mark Dimensions to Identify Scavengers. J. Forensic Sci..

[57] Pokines J.T. (2015). Taphonomic alterations by the rodent species woodland vole (*microtus pinetorum*) upon human skeletal remains. Forensic Sci. Int..

[58] Schulz I., Schneider P.M., Olek K., Rothschild M.A., Tsokos M. (2006). Examination of postmortem animal interference to human remains using cross-species multiplex pcr. Forensic Sci. Med. Pathol..

[59] Binford L.R. (1987). Bones Ancient Men and Modern Myths.

[60] Hall C.M., Bryant K.A., Haskard K., Major T., Bruce S., Calver M.C. (2016). Factors determining the home ranges of pet cats: A meta-analysis. Biol. Conserv..

[61] Natural Biodiversity Network (NBN) (2021). *NBN Atlas*; NBN Atlas Partnership. https://nbnatlas.org/.

[62] Kleiman D.G., Geist V., McDade M.C. (2004). Grzimek’s Animal Life Encyclopedia.

[63] Olsen L.H. (2013). Tracks and Signs of the Animals and Birds of Britain and Europe.

[64] Ragg J.R., Mackintosh C.G., Moller H. (2000). The Scavenging Behaviour of Ferrets (mustela furo), Feral Cats (felis domesticus), Possums (trichosurus vulpecula), Hedgehogs (erinaceus europaeus) and Harrier Hawks (circus approximans) on Pastoral Farmland in New Zealand: Implications for Bovine Tuberculosis Transmission. N. Z. Vet. J..

[65] Rossi M.L., Shahrom A.W., Chapman R.C., Vanezis P. (1994). Postmortem injuries by indoor pets. Am. J. Forensic Med. Pathol..

[66] Suntirukpong A., Mann R.W., DeFreytas J.R. (2017). Postmortem scavenging of human remains by domestic cats. Siriraj Med. J..

[67] Garcia S., Smith A., Baigent C., Connor M. (2020). The Scavenging Patterns of Feral Cats on Human Remains in an Outdoor Setting. J. Forensic Sci..

[68] Byard R.W. (2020). Postmortem Predation by a Clowder of Domestic Cats. Forensic Sci. Med. Pathol..

[69] Sperhake J.P., Tsokos M. (2001). Postmortem animal depredation by a domestic cat. Arch. Kriminol..

[70] Prahlow J.A., Byard R.W. (2012). Atlas of Forensic Pathology.

[71] Byard R.W. (2020). An Unusual Pattern of Post-Mortem Injury Caused by Australian Fresh Water Yabbies (cherax destructor). Forensic Sci. Med. Pathol..

[72] Bauer J.W., Logan K.A., Sweanor L.L., Boyce W.M., Jones C.A. (2005). Scavenging behavior in puma. Southwest. Nat..

[73] Vander Wall S.B. (1990). Food Hoarding in Animals.

[74] Bischoff-Mattson Z., Mattson D. (2009). Effects of simulated mountain lion caching on decomposition of ungulate carcasses. West. N. Am. Nat..

[75] Moran N.C., O’Connor T.P. (1991). Bones that cats gnawed upon. Circaea.

[76] Álvarez M.C., Kaufmann C.A., Massigoge A., Gutiérrez M.A., Rafuse D.J., Scheifler N.A., González M.E. (2012). Bone Modification and Destruction Patterns of Leporid Carcasses by Geoffroy’s Cat (Leopardus Geoffroyi): An Experimental Study. Quat. Int..

[77] Scott D.M., Berg M.J., Tolhurst B.A., Chauvenet A.L., Smith G.C., Neaves K., Lochhead J., Baker P.J. (2014). Changes in the Distribution of Red Foxes (*Vulpes Vulpes*) in Urban Areas in Great Britain: Findings and Limitations of a 134 Media-Driven Nationwide Survey. PLoS ONE.

[78] Kostecke R.M., Linz G.M., Bleier W.J. (2001). Survival of avian carcasses and photographic evidence of predators and scavengers. J. Field Ornithol..

[79] De Vault T.L., Rhodes O.E. (2002). Identification of vertebrate scavengers of small mammal carcasses in a forested landscape. Acta Theriol..

[80] Selva N., Jędrzejewska B., Jędrzejewski W., Wajrak A. (2005). Factors Affecting Carcass Use by a Guild of Scavengers in European Temperate Woodland. Can. J. Zool..

[81] Junod C.A. (2013). Subaerial Bone Weathering and other Taphonomic Changes in a Temperate Climate. Master’s Thesis.

[82] Olson Z.H., Beasley J.C., Rhodes O.E. (2016). Carcass Type Affects Local Scavenger Guilds More than Habitat Connectivity. PLoS ONE.

[83] Schumann M., Nolte I., Huckenbeck W., Barz J. (1996). Tierfrass—Wenige stunden nach todeseintritt. Rechtsmedizin.

[84] Tsokos M., Schultz F. (1999). Indoor postmortem animal interference by carnivores and rodents—Report of two cases and review of the literature. Int. J. Leg. Med..

[85] Romain N., Brandt-Casadevall C., Dimo-Simonin K.M., Mangin P., Papilloud J. (2002). Post-mortem castration by a dog—A case report. Med. Sci. Law.

[86] Steadman D.W., Worne H. (2007). Canine Scavenging of Human Remains in an Indoor Setting. Forensic Sci. Int..

[87] Verzeletti A., Cortellini V., Vassalini M. (2010). Post-Mortem Injuries by a Dog: A Case Report. J. Forensic Leg. Med..

[88] Colard T., Delannoy Y., Naji S., Gosset D., Hartnett K., Becart A. (2015). Specific Patterns of Canine Scavenging in Indoor Settings. J. Forensic Sci..

[89] Hernández-Carrasco M., Pisani J.M.A., Scarso-Giaconi F., Fonseca G.M. (2016). Indoor postmortem mutilation by dogs: Confusion, contradictions, and needs from the perspective of the forensic veterinarian medicine. J. Vet. Behav..

[90] Hewson R., Kolb H.H. (1976). Scavenging on sheep carcases by foxes (vulpes vulpes) and badgers (meles meles). Notes Mammal Soc..

[91] Kaczensky P., Hayes R.D., Promberger C. (2005). Effect of raven corvus corax scavenging on the kill rates of wolf canis lupus packs. Wildl. Biol..

[92] Reed E.H. (2001). Disarticulation of kangaroo skeletons in semi-arid Australia. Aust. J. Zool..

[93] Morton R.J., Lord W.D. (2006). Taphonomy of Child-Sized Remains: A study of Scattering and Scavenging in Virginia, USA. J. Forensic Sci..

[94] Brown O.J.F., Field J., Letnic M. (2006). Variation in the taphonomic effect of scavengers in semi-arid Australia linked to rainfall and the el niño southern oscillation. Int. J. Osteoarchaeol..

[95] Haglund W.D., Reay D.T., Swindler D.R. (1989). Canid Scavenging Disarticulation Sequence of Human Remains in the Pacific Northwest. J. Forensic Sci..

[96] Young A., Marquez-Grant N., Stillman R., Smith M.J., Korstjens A.H. (2014). An Investigation of Red Fox (vulpes vulpes) and Eurasian Badger (meles meles) Scavenging, Scattering, and Removal of Deer Remains: Forensic Implications and Applications. J. Forensic Sci..

[97] Gadbois S., Sievert O., Reeve C., Harrington F.H., Fentress J.C. (2015). Revisiting the Concept of Behavior Patterns in Animal Behavior with an Example from Food-Caching Sequences in Wolves (canis lupus), Coyotes (canis latrans), and Red Foxes (vulpes vulpes). Behav. Process..

[98] D’Andrea A.C., Gotthardt R.M. (1984). Predator and scavenger modification of recent equid skeletal assemblages (wolves). ARCTIC.

[99] Haglund W.D., Reay D.T., Swindler D.R. (1988). Tooth mark artifacts and survival of bones in animal scavenged human skeletons. J. Forensic Sci..

[100] Pokines J. (2015). A procedure for processing outdoor surface forensic scenes yielding skeletal remains among leaf litter. J. Forensic Identif..

[101] Mech L.D., Boitani L. (2003). Wolves: Behavior, Ecology, and Conservation.

[102] De Munnynck K., van de Voorde W. (2002). Forensic Approach of Fatal Dog Attacks: A Case Report and Literature Review. Int. J. Leg. Med..

[103] Haynes G. (1980). Evidence of carnivore gnawing on pleistocene and recent mammalian bones. Pelobiology.

[104] Haynes G. (1983). A guide for differentiating mammalian carnivore taxa responsible for gnaw damage to herbivore limb bones. Paleobiology.

[105] Pickering T.R. (2001). Carnivore voiding—A taphonomic process with the potential for the deposition of forensic evidence. J. Forensic Sci..

[106] Esteban-Nadal M., Cáceres I., Fosse P. (2010). Characterization of a Current Coprogenic Sample Originated by Canis Lupus as a Tool for Identifying a Taphonomic Agent. J. Archaeol. Sci..

[107] Bright L.N. (2011). Taphonomic Signatures of Animal Scavenging in Northern California—A Forensic Anthropological Analysis. Master’s Thesis.

[108] Sala N., Arsuaga J.L. (2013). Taphonomic Studies with Wild Brown Bears (Ursus Arctos) in the Mountains of Northern Spain. J. Archaeol. Sci..

[109] Elgmork K. (1982). Caching behaviour of brown bears. J. Mammal..

[110] Saladié P., Huguet R., Diez C., Rodriguez-Hidalgo A., Carbonell E. (2013). Taphonomic modifications produced by modern brown bears (ursus arctos). Int. J. Osteoarchaeol..

[111] Carson E.A., Stefan H.V., Powell J.F. (2000). Skeletal manifestations of bear scavenging. J. Forensic Sci..

[112] Udoni M. (2017). A Taphonomic Study of Black Bear (Ursus Americanus) and Grizzly Bear (u. Arctos) Tooth Marks on Bone. Master’s Thesis.

[113] Macdonald R.A. (2002). Resource partitioning among British and Irish mustelids. J. Anim. Ecol..

[114] Lee S., Mill P.J. (2004). Cranial Variation in British Mustelids. J. Morphol..

[115] Ryšavá-Nováková M., Koubek P. (2009). Feeding habits of two sympatric mustelid species, European polecat and stone marten in the Czech Republic. Folia Zool..

[116] Hobischak N.R. (1997). Freshwater Invertebrate Succession and Decompositional Studies on Carrion in British Columbia. Master’s Thesis.

[117] King K.A., Lord W.D., Ketchum H.R., O’Brien R.C. (2016). Postmortem Scavenging by the Virginia Opossum (didelphis virginiana): Impact on Taphonomic Assemblages and Progression. Forensic Sci. Int..

[118] Pokines J., Pollock C. (2018). The Small Scavenger Guild of Massachusetts. Forensic Anthropol..

[119] MacDonnel N., Anderson G. (1997). Aquatic Forensics: Determination of Time since Submergence Using Aquatic Invertebrates.

[120] Kruuk H. (1978). Spatial Organization and Territorial Behaviour of the European Badger (*Meles Meles*). J. Zool..

[121] Young A., Schotsmans E.M.J., Márquez-Grant N., Forbes S.L. (2017). The effects of terrestrial mammalian scavenging and avian scavenging on the body. Taphonomy of Human Remains: Forensic Analysis of the Dead and the Depositional Environment.

[122] Wroe S., McHenry C., Thomason J. (2005). Bite club—Comparative bite force in big biting mammals and the prediction of predatory behaviour in fossil taxa. Biol. Sci..

[123] Jeong Y., Jantz L.M., Smith J. (2016). Investigation into Seasonal Scavenging Patterns of Raccoons on Human Decomposition. J. Forensic Sci..

[124] Hannigan A. (2015). A Descriptive Study of Forensic Implications of Raccoon Scavenging in Maine.

[125] Berryman H.E., Sorg M.H., Haglund W.D. (2002). Disarticulation pattern and tooth mark artifacts associated with pig scavenging of human remains: A case study. Advances in Forensic Taphonomy: Method, Theory, and Archaeological Perspectives.

[126] De Vault T.L., Brisbin J.I.L., Rhodes J.O.E. (2004). Factors influencing the acquisition of rodent carrion by vertebrate scavengers and decomposers. Can. J. Zool..

[127] Kleiman D.G., Geist V., McDade M.C., Mammals I.V. (2004). Grzimek’s Animal Life Encyclopedia.

[128] Tucak Z. (1996). Ergebnisse von 155 mageninhaltsuntersuchungen von schwarzwild (*sus scrofa l.*) im ungegatterten teil des waldjagdrevieres belje in Baranja. Z. Jagdwiss..

[129] Greenfield H.J. (1988). Bone Consumption by Pigs in a Contemporary Serbian Village. J. Field Rchaeol..

[130] Domínguez-Solera S.D., Domínguez-Rodrigo M. (2009). A taphonomic study of bone modification and of tooth-mark patterns on long limb bone portions by suids. Int. J. Osteoarchaeol..

[131] Ropohl D., Scheithauer R., Pollak S. (1995). Postmortem injuries inflicted by domestic golden hamster—Morphological aspects and evidence by DNA typing. Forensic Sci. Int..

[132] Erkol Z., Hösükler E. (2018). Postmortem animal attacks on human corpses. Post Mortem Examination and Autopsy—Current Issues from Death to Laboratory Analysis.

[133] Bumann G.B., Stauffer D.F. (2002). Scavenging of Ruffed Grouse in the Appalachians Influences and Implications. Wildl. Soc. Bull..

[134] Pokines J.T., Sussman R., Gough M., Ralston C., McLeod E., Brun K., Kearns A., Moore T.L. (2017). Taphonomic Analysis of Rodentia and Lagomorpha Bone Gnawing Based upon Incisor Size. J. Forensic Sci..

[135] Haglund W.D., Sorg M.H., Haglund W.D. (1997). Rodents and human remains. Forensic Taphonomy: The Postmortem Fate of Human Remains.

[136] Keyes C.A., Myburgh J., Brits D. (2020). Scavenger activity in a peri-urban agricultural setting in the highveld of South Africa. Int. J. Leg. Med..

[137] Sutcliffe A.J. (1973). Similarity of bones and antlers gnawed by deer to human artefacts. Nature.

[138] Kierdorf U. (1994). A further example of long-bone damage due to chewing by deer. Int. J. Osteoarchaeol..

[139] Brothwell D. (1976). Further evidence of bone chewing by ungulates—The sheep of North Ronaldsay, Orkney. J. Archaeol. Sci..

[140] Meckel L.A., McDaneld C.P., Wescott D.J. (2018). White-Tailed Deer as a Taphonomic Agent: Photographic Evidence of White-Tailed Deer Gnawing on Human Bone. J. Forensic Sci..

[141] Cáceres I., Esteban-Nadal M., Bennàsar M., Fernández-Jalvo Y. (2011). Was it the deer or the fox?. J. Archaeol. Sci..

[142] Johnson D.L., Haynes G. (1985). Camels as taphonomic agents. Quat. Res..

[143] Haynes G. (1983). Frequencies of spiral and green-bone fractures on ungulate limb bones in modern surface assemblages. Am. Antiq..

[144] Behrensmeyer A.K., Gordon K.D., Yanagi G.T. (1986). Trampling as a cause of bone surface damage and pseudo-cutmarks. Nature.

[145] Gifford-Gonzalez D. (2018). An Introduction to Zooarchaeology.

[146] Magoun A.J. Summer scavenging activity in Northeastern Alaska. Proceedings of the Conference on Scientific Research in the National Parks.

[147] France D.L., Griffin T.J., Swanburg J.G., Lindemann J.W., Davenport G.C., Trammell V., Travis C.T., Kondratieff B., Nelson A., Castellano K., Sorg M.H., Haglund W.D. (1997). Necrosearch revisited: Further multidisciplinary approaches to the detection of clandestine graves. Forensic Taphonomy: The Postmortem Fate of Human Remains.

[148] Demo C., Cansi E.R., Kosmann C., Pujol-Luz J.R. (2013). Vultures and others scavenger vertebrates associated with man-sized pig carcasses: A perspective in forensic taphonomy. Zoologia.

[149] Fetner R.A., Sołtysiak A. (2013). Shape and distribution of griffon vulture (gyps fulvus) scavenging marks on a bovine skull. J. Taphon..

[150] Pharr L. Comparison of vulture scavenging rates at the Texas state forensic anthropology research facility versus off-site, non-forensic locations. Proceedings of the American Academy of Forensic Sciences 64th Annual Scientific Conference.

[151] Lewis K.N. (2018). The Effects of Clothing on Vulture Scavenging and Spatial Distribution of Human Remains in Central Texas.

[152] Halley D.J., Gjershaug J.O. (1998). Inter- and intra-specific dominance relationships and feeding behaviour of eagles at carcasses. IBIS.

[153] Sanders W.J., Trapani J., Mitani J.C. (2003). Taphonomic aspects of crowned hawk-eagle predation on monkeys. J. Hum. Evol..

[154] Trapani J., Sanders W.J., Mitani J.C., Heard A. (2006). Precision and consistency of the taphonomic signature of predation by crowned hawk-eagles (stephanoaetus coronatus) in Kibale National Park, Uganda. Palaios.

[155] Hewson R. (1981). Scavenging of mammal carcases by birds in West Scotland. J. Zool..

[156] Asamura H., Takayanagi K., Ota M., Kobayashi K., Fukushima H. (2004). Unusual characteristic patterns of postmortem injuries. J. Forensic Sci..

[157] Young A. (2013). An Investigation of Patterns of Mammalian Scavenging in Relation to Vertebrate Skeletal Remains. Ph.D. Thesis.

[158] Dettling A., Strohbeck-Kühner P., Schmitt G., Haffner H.T. (2001). Tierfrass durch einen singvogel?. Archiv. Kriminol..

[159] Pokines J.T. (2022). Preliminary Study of Gull (Laridae) Scavenging and Dispersal of Vertebrate Remains, Shoals Marine Laboratory, Coastal New England. J. Forensic Sci..

[160] Klein A. (2013). Vulture Scavenging of Pig Remains at Varying Grave Depths. Master’s Thesis.

[161] Ray R.R., Seibold H., Heurich M. (2014). Invertebrates Outcompete Vertebrate Facultative Scavengers in Simulated Lynx Kills in the Bavarian Forest 132 National Park, Germany. Anim. Biodivers. Conserv..

[162] Williams A., Rogers C.J., Cassella J.P. (2019). Why does the UK Need a Human Taphonomy Facility?. Forensic Sci. Int..

[163] Pecsi E.L., Bronchti G., Crispino F., Forbes S.L. (2020). Perspectives on the Establishment of a Canadian Human Taphonomic Facility: The Experience of Rest(ES). Forensic Sci. Int. Synerg..

[164] Cockle D.L., Bell L.S. (2019). The impact of trauma and blood loss on human decomposition. Sci. Justice.

[165] Spies M.J., Finaughty D.A., Friedling L.J., Gibbon V.E. (2020). The Effect of Clothing on Decomposition and Vertebrate Scavengers in Cooler Months of the Temperate Southwestern Cape, South Africa. Forensic Sci. Int..

[166] Roberts L.G., Dabbs G.R. (2015). A Taphonomic Study Exploring the Differences in Decomposition Rate and Manner between Frozen and never Frozen Domestic Pigs (Sus Scrofa). J. Forensic Sci..

[167] Kaufmann C.A., Rafuse D.J., González M.E., Álvarez M.C., Massigoge A., Scheifler N.A., Gutiérrez M.A. (2016). Carcass Utilization and Bone Modifications on Guanaco Killed by Puma in Northern Patagonia, Argentina. Quat. Int..

[168] Sorg M.H. (2019). Differentiating Trauma from Taphonomic Alterations. Forensic Sci. Int..

[169] Cattaneo C., Cappella A., Schotsmans E.M.J., Márquez-Grant N., Forbes S.L. (2017). Distinguishing between peri- and post-mortem trauma on bone. Taphonomy of Human Remains. Forensic Analysis of the Dead and the Depositional Environment: Forensic Analysis of the Dead and the Depositional Environment.

[170] Bass W.M., Driscoll P.A. (1983). Summary of Skeletal Identification in Tennessee: 1971–1981. J. Forensic Sci..

[171] Love J.C. (2019). Sharp force trauma analysis in bone and cartilage: A literature review. Forensic Sci. Int..

[172] Gunawardena S.A. (2016). Artefactual Incised Wounds due to Postmortem Predation by the Sri Lankan Water Monitor (Kabaragoya). Forensic Sci. Med. Pathol..

